# Long non-coding RNA H19 confers 5-Fu resistance in colorectal cancer by promoting SIRT1-mediated autophagy

**DOI:** 10.1038/s41419-018-1187-4

**Published:** 2018-11-19

**Authors:** Meng Wang, Dong Han, Ziming Yuan, Hanqing Hu, Zhixun Zhao, Runkun Yang, Yinghu Jin, Chaoxia Zou, Yinggang Chen, Guiyu Wang, Xu Gao, Xishan Wang

**Affiliations:** 1 Department of Colorectal Surgery, the Second Affiliated Hospital of Harbin Medical University, Harbin, 150081 China; 2 Department of Biochemistry and Molecular Biology, Harbin Medical University, Harbin, 150081 China; 3 Department of Colorectal Surgery, Cancer Institute and Hospital, Chinese Academy of Medical Sciences, Peking Union Medical College, Beijing, 100000 China

## Abstract

Chemotherapy failure is the major cause of recurrence and poor prognosis in colorectal cancer (CRC) patients. The role of the differentially expressed lncRNAs in 5-Fluorouracil chemoresistance has not fully explained. Here, we observed lncRNA H19 was associated with the 5-Fu resistance in CRC. Quantitative analysis indicated that H19 was significantly increased in recurrent CRC patient samples. Kaplan–Meier survival analysis indicated that high H19 expression in CRC tissues was significantly associated with poor recurrent free survival. Our functional studies demonstrated that H19 promoted colorectal cells 5-Fu resistance. Mechanistically, H19 triggered autophagy via SIRT1 to induce cancer chemoresistance. Furthermore, bioinformatics analysis showed that miR-194–5p could directly bind to H19, suggesting H19 might work as a ceRNA to sponge miR-194–5p, which was confirmed by Dual-luciferase reporter assay and Immunoprecipitation assay. Extensively, our study also showed that SIRT1 is the novel direct target of miR-194–5p in CRC cells. Taken together, our study suggests that H19 mediates 5-Fu resistance in CRC via SIRT1 mediated autophagy. Our finding provides a novel mechanistic role of H19 in CRC chemoresistance, suggesting that H19 may function as a marker for prediction of chemotherapeutic response to 5-Fu.

## Introduction

Colorectal cancer (CRC) is a common human malignancy as the third leading cause of cancer-related death worldwide and characterized by poor prognosis and treatment^1^. Although many novel drugs have been developed for patients with advanced CRC, 5-fluorouracil (5-Fu) is still widely used as the classic and basic drugs in adjuvant chemotherapy and palliative chemotherapy, which has been written in the main CRC treatment guidelines. 5-Fu targets thymidylate synthase to exert anticancer effects through blocking the normal synthesis of DNA and disrupting RNA processing^2^. However, It has been reported that some CRC patients are primarily resistant to 5-Fu-based chemotherapy, while some will acquire the resistance, afterwards^3,4^. Thus, it is necessary to reveal the potential targets for treatment of CRC patients with 5-Fu resistance. Recently, therapy-induced autophagy was shown to be a novel mechanism of resistance to anticancer agents^5^. Autophagy supports the survival of tumor cells under metabolic and therapeutic stress^6^ by sequestering organelles and proteins in autophagic vesicles and delivering cytoplasmic cargo to lysosomes for degradation^7^. Some autophagy inhibitors have been shown to enhance the efficacy of chemotherapy for various cancers. For example, the autophagy inhibitor CQ has been applied to treat glioblastoma and breast cancer^5,8^. Consequently, to target the switch between autophagy and apoptosis may be a promising strategy for adjuvant chemotherapy in CRC.

lncRNAs belong to a kind of non-coding RNA transcripts, lacking in protein-coding capacity. It is well known that dysregulated lncRNAs plays a crucial role in cancers^9^. Previously we reviewed recent findings of emerging roles for lncRNAs in CRC and discuss rapid translational lncRNA research on clinical application in diagnosis, prognosis, and potential treatment^10^. Some lncRNAs have been proved to play an important role in the tumor drug resistance^11^, such as lncRNA MIR100HG which mediates cetuximab resistance via Wnt/beta-catenin signaling^12^. H19 promotes 5-Fu resistance in osteosarcoma by increasing the expression of FOXC2^13^. lncRNA HULC triggers autophagy attenuates the chemosensitivity of HCC cells^14^. Exosome-transmitted lncARSR promotes sunitinib resistance in renal cancer by acting as a competing endogenous RNA^15^. To date, the research on lncRNA in CRC 5-Fu resistance is quite limited^16^. There’re very few studies analyzing in-depth this issue. LncRNA H19 gene is located on chromosome 11 in human and is a maternally expressed imprinted gene that plays a vital role in mammalian development^17^. The pathological function of H19 as a non-coding RNA in tumor is recently being elucidated, although H19 has been intensively studied in epigenetic. Much evidence shows that H19 is upregulated in bladder cancer^18^, breast cancer^19^, and CRC^20,21^. Previously, we found H19 indicated a poor prognosis of colorectal cancer and promoted tumor growth by recruiting and binding to eIF4A3^22^. However, the biological role of H19 on CRC cell 5-Fu chemoresistance remains poorly understood.

In this study, we first showed that H19 was up-regulated in CRC recurrent patients and correlated with recurrent free survival (RFS). Functional analyses showed that H19 could enhance 5-Fu chemoresistance in CRC cells. Mechanistic studies demonstrated that H19 promoted autophagy via SIRT1. Besides, H19 worked as a competitive endogenous RNA (ceRNA) of miR-194–5p, which is a suppressive microRNA (miRNA) of SIRT1. The present work reveals a novel regulatory pathway of H19/miR-194–5p/SIRT1/autophagy in 5-Fu resistance of CRC cells, suggesting that H19 is a new prognostic factor and potential therapeutic target in CRC.

## Results

### H19 is associated with colorectal cancer recurrence and patient outcome

**Fig. 1:**
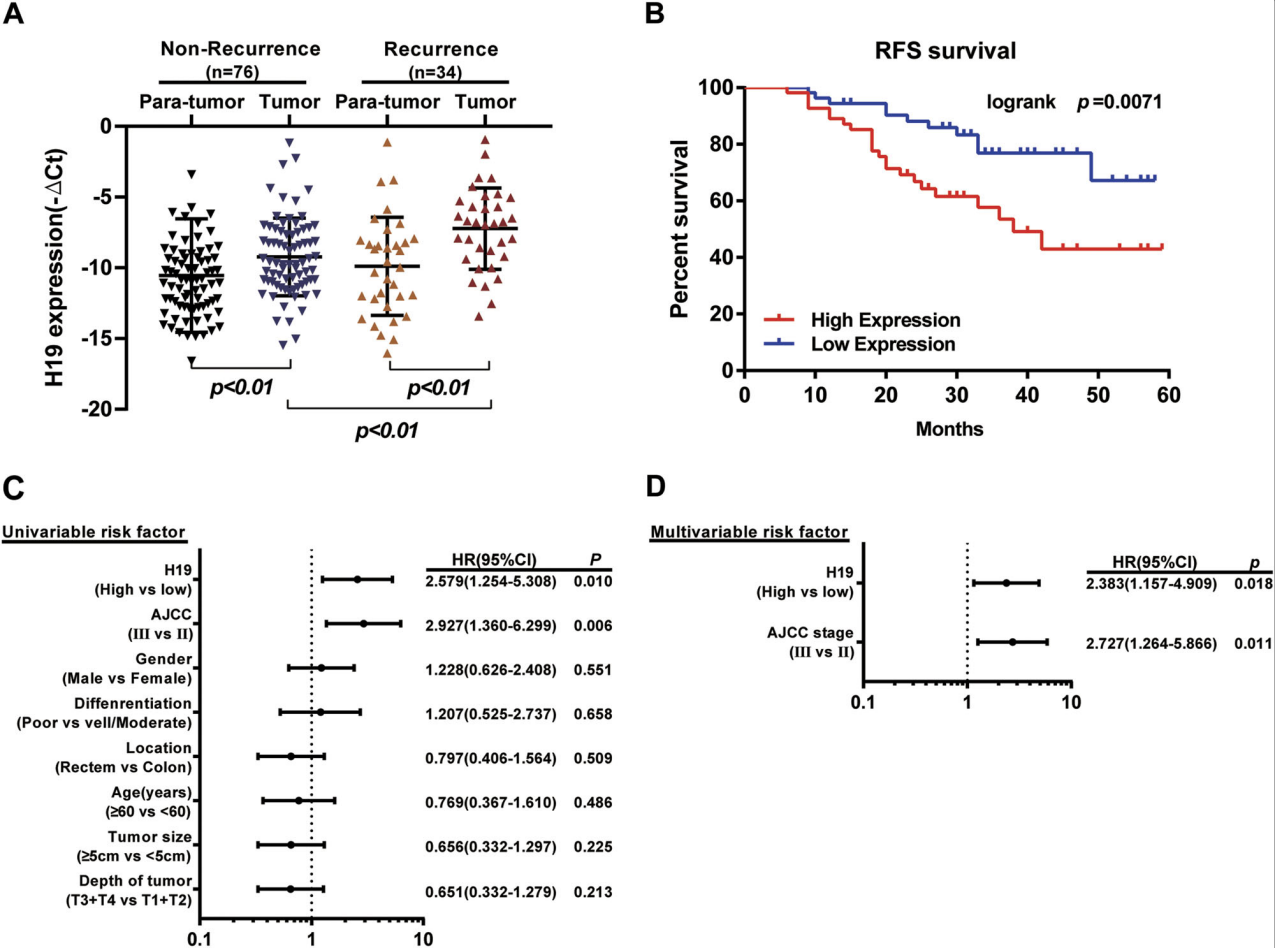
Elevated lncRNA H19 was expressed in CRC tissues and cell lines. **a** Quantitative RT-PCR (qRT-PCR) analysis of H19 expression in CRC tissues and pair-matched adjacent normal samples (Non-recurrence group *n* = 76, and Recurrence group *n* = 34). **b** Kaplan–Meier analyses of the associations between H19 expression level and recurrence free survival (RFS) of patients with CRC (the log-rank test was used to calculate *P*-values). *P* = 0.0071 vs. low H19 expression group. **c** Univariate analysis was performed in previous cohort. The bars correspond to 95% confidence intervals. **d** Multivariate analysis was performed in previous cohort. The bars correspond to 95% confidence intervals

To study the role of H19 in CRC, we first detected its expression in 110 paired CRC tissues and para-tumor tissues. Clinicopathological features were summarized in Supplementary Table S1. The detailed relationships between the H19 expression status and clinicopathological variables of 110 patients are shown in Supplementary Table S2. Noticeably, high expression of H19 in CRC had a significant correlation with the tumor differentiation (*P* = 0.023) and depth of tumor (*P* = 0.007). Real-time PCR showed that H19 was obviously upregulated in 34 CRC tissues from patients with recurrence (*P* <0.001, Fig. 1a). This suggests that H19 may play a role in CRC recurrence.

The survival analysis showed that patients in the H19-high-expression group showed a shorter recurrent free survival (RFS) than those in the H19-low-expression group (*P* = 0.0071, Fig. 1b). Furthermore, univariate (Fig. 1c) and multivariate Cox regression analyses (hazard ratio (HR) = 2.383, 95% confidence interval (CI) = 1.157–4.909, *P* = 0.018, Fig. 1d) further identified that H19 was an independent prognosis factor for CRC aggressiveness with significant hazard ratios for predicting clinical outcome. Its predictive value was comparable to that of the AJCC stage. Thus, the data define the potential value of the amount of H19 in predicting CRC recurrence, indicating that patients with higher H19 expression levels have higher risks of CRC recurrence.

### H19 promotes 5-Fu resistance in colorectal cancer cells

**Fig. 2:**
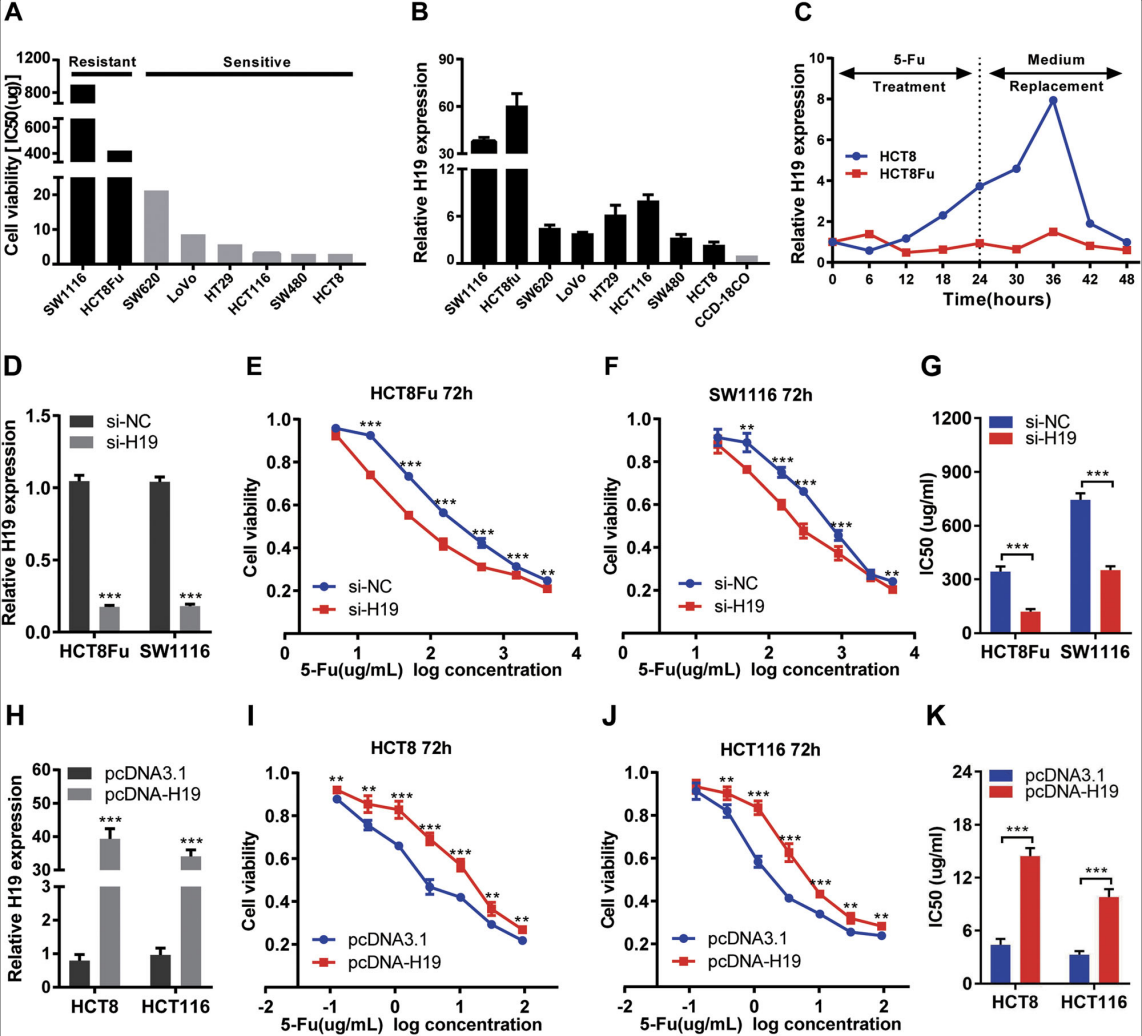
H19 promotes 5-Fu resistance in CRC cells. **a** The cytotoxic effect of 5-Fu in a panel of colorectal cell lines was measured by MTT assay after 72 h treatment with 5-Fu and IC50 value was calculated. **b** Relative expression of H19 in CRC cell lines and the normal colon epithelium cell line CCD-18Co by qRT-PCR. Data are presented as mean±SD from three independent experiments. **c** Dynamic changes of H19 expression level in response to 5-Fu treatment (0.1 μg/ml). The 1–8 time courses successively present 6, 12, 18, 24h after 5-Fu treatment and 30, 36, 42, 48h after medium replacement. **d**, **h** Validation of knockdown and overexpression efficacy of H19 in CRC cell line by qRT-PCR. ***P<0.001. **e**, **f** The cell sensitivity of HCT8Fu and SW1116 cells transfected with si-NC or si-H19, **i**, **j** HCT8 and HCT116 transfected with pcDNA3.1 or pcDNA-H19 to 5-Fu was evaluated using the MTT assay upon exposure to the step-up concentration of 5-Fu for 72h. **P<0.01, ***P<0.001. **g**, **k** Effects of H19 knockdown and overexpression on the IC50 value of cells was calculated. ***P<0.001

To characterize the sensitivity of CRC cells to 5-Fu treatment, a panel of CRC cells was treated with various concentrations of 5-Fu for 3 days, including one pair of 5-Fu resistance CRC cells (HCT8Fu) and their parental 5-Fu-sensitive cells (HCT8), and then the MTT assay was performed. Considering that the HCT8Fu was generated in a stepwise manner by exposing drug-sensitive HCT8 cells to increasing doses of 5-Fu, cells with IC50 values above 400 μg/ml were regarded as 5-Fu resistant. According to this criterion, another cell line SW1116 was identified as primary 5-Fu resistant (Fig. 2a). QRT-PCR showed that H19 expression was obviously upregulated both in primary 5-Fu resistant SW1116 cell line and acquired 5-Fu resistant HCT8Fu cell line (Fig. 2b). In addition, H19 was induced by 5-Fu treatment in drug-sensitive cells, but not in drug-resistant cell lines. H19 expression increased significantly 24h after drug treatment in the drug-sensitive cell line and reached its peak 12h after replacement of the culture medium (Fig. 2c), indicating that H19 was involved in the response to 5-Fu treatment.

To investigate the biological functions of H19 in the chemoresistance of CRC against 5-Fu, we performed MTT assay to examine the effect of H19 on cell sensitivity to anticancer drugs in CRC cells. 5-Fu resistant HCT8Fu and SW1116 cells were transfected H19-specific siRNA, and control siRNA served as a negative control. 5-Fu sensitive HCT8 and HCT116 cells were transfected with the H19 expression vector or empty vector. The validation of knockdown and overexpression efficacy of H19 was evaluated by qRT-PCR (Fig. 2d, h). IC50 value of 5-Fu in response to H19 down or up-regulation was measured. Compared with HCT8Fu (or SW1116) cells transfected with si-NC, the IC50 value of 5-Fu in cells transfected with si-H19 was reduced by 64.94% (or 52.72%) (*P* < 0.001, *P*<0.001, Fig. 2g). Compared with HCT8 (or HCT116) cells transfected with empty vector, the IC50 value of 5-Fu in cells transfected with H19 was increased by 227.43% (or 198.69%) (*P*<0.001, *P*<0.001, Fig. 2k).

**Fig. 3:**
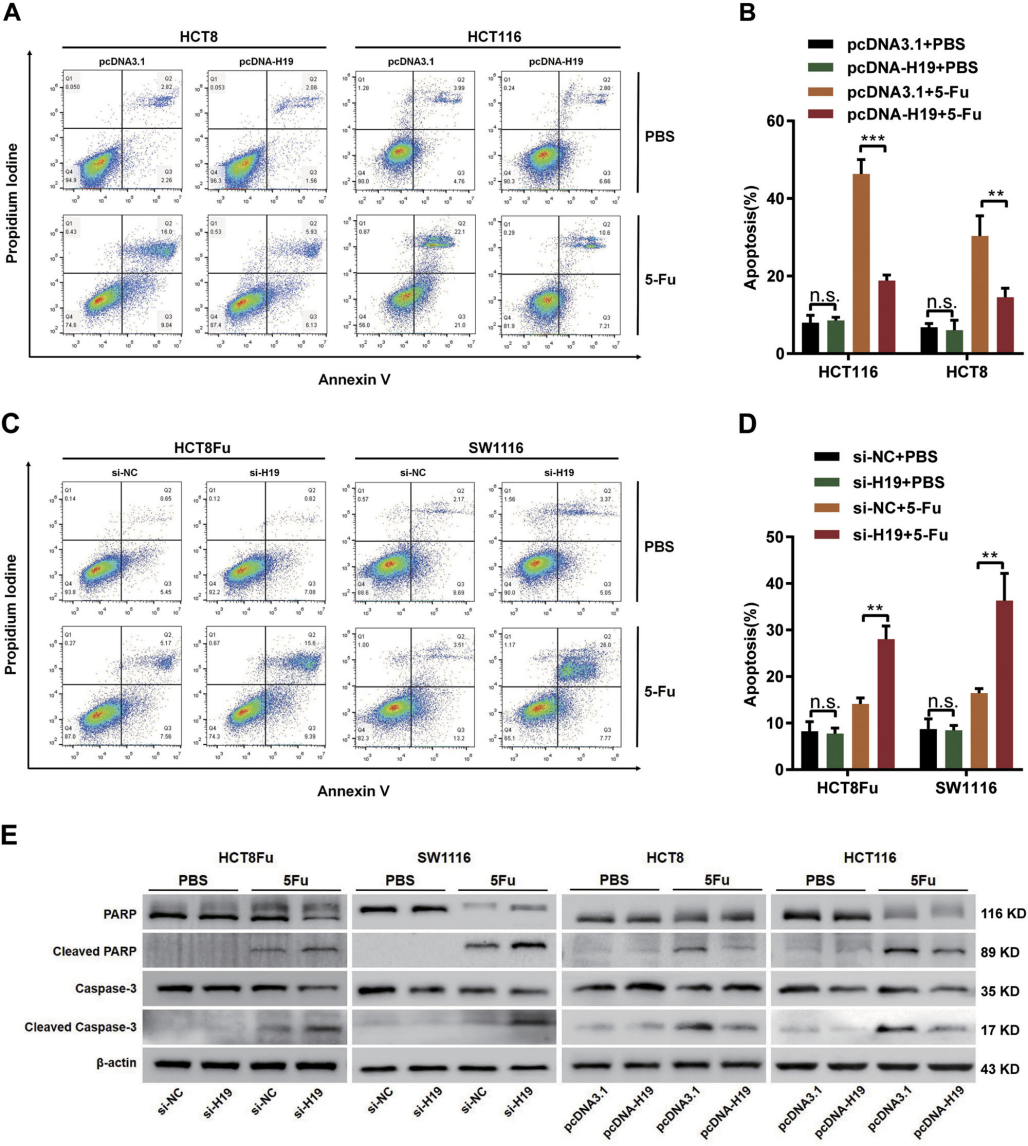
H19 reduces apoptosis by chemotherapeutic agents 5-Fu. **a**, **c** Apoptosis was detected by flow cytometry in HCT8, HCT116 cells transfected with pcDNA3.1 or pcDNA-H19 (**a**) and HCT8Fu, SW1116 cells transfected with si-NC or si-H19 (**c**). The cells were treated with PBS and 5-Fu. **b**, **d** Columns are the average of three independent experiments. Data are presented as mean±SD from three independent experiments. **P<0.01, ***P<0.001. **e** Caspase-3, PARP, and their cleaved forms were detected by western blotting

To examine whether the effect of H19 on cells 5-Fu sensitivity reflects changes in the apoptosis, we performed flow cytometry analysis by staining cells with annexin V-FITC and PI. However, H19 did not affect cell apoptosis after the transfection of either si-H19 or pcDNA-H19 without 5-Fu treatment. As for 5-Fu sensitive HCT8 and HCT116 cells, overexpression H19 decreased the cell apoptosis only in 5-Fu treatment group by flow cytometry (Fig. 3a, b). Additionally, 5-Fu resistant HCT8Fu and SW1116 cells exhibited marked changes in response to 5-Fu treatment when H19 was knocked down and increased the cell apoptosis only in 5-Fu treatment group (Fig. 3c, d). Consistently, western blotting assay showed changes cleaved caspase-3 and cleaved PARP caused by 5-Fu (Fig. 3e). To further confirm the role of H19, we carried out the survival experiment using siRNA resistant H19 construct, and then detect the cell viability (Fig. SA, SB) and apoptosis (Fig. SC, SD). The results showed a rescue effect of cell death. However, MTT assays and flow cytometric analysis did not reveal functions of H19 in the chemoresistance of CRC against oxaliplatin (Fig. SE to SJ). Taken together, these results imply that H19 enhances resistance to 5-Fu via reducing cell death only under 5-Fu stress.

### H19 induces cancer chemoresistance via the autophagy pathway

**Fig. 4:**
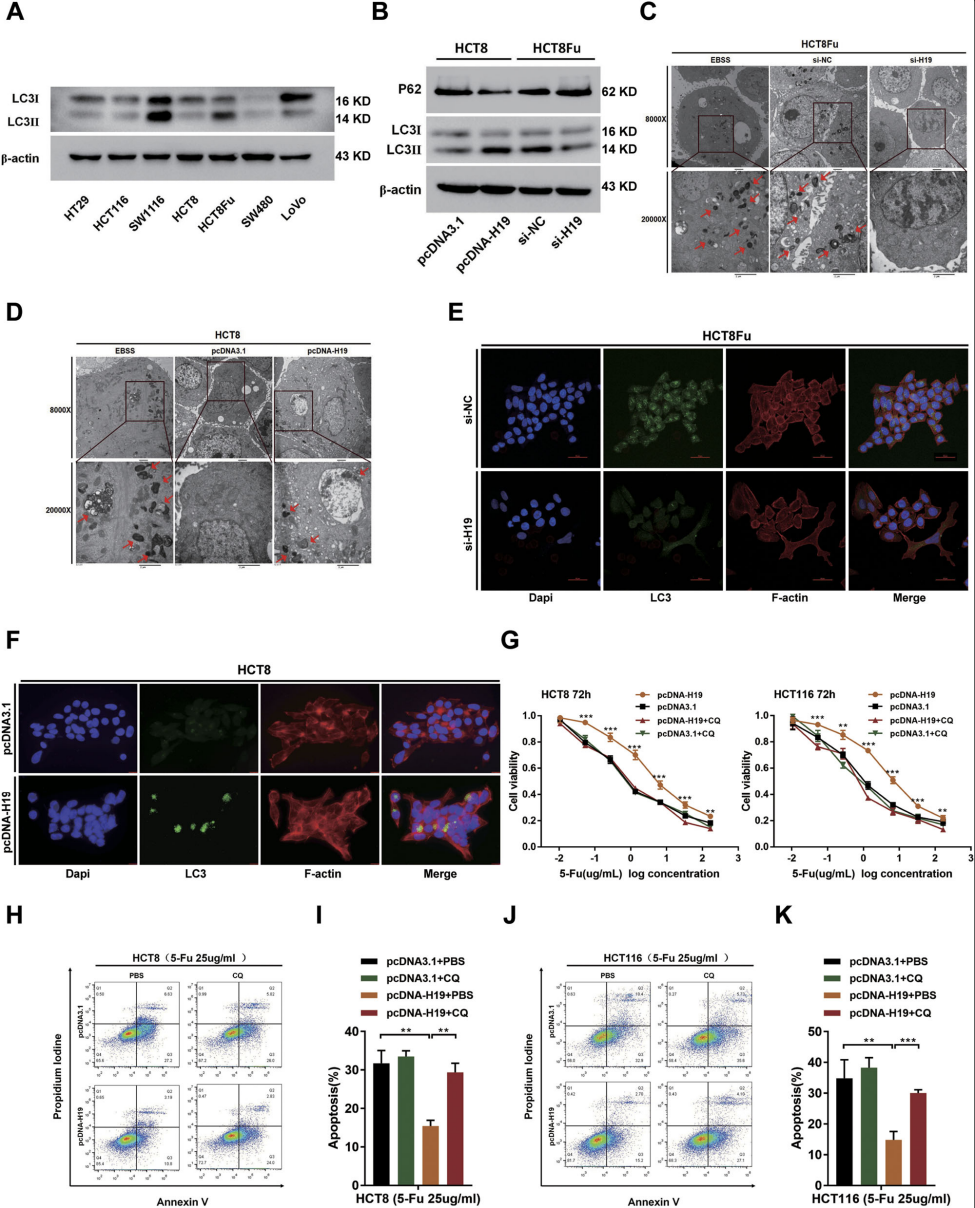
H19 activates cancer autophagy to mediate 5-Fu resistance. **a** The basic levels of LC3 protein in CRC cell lines were detected by western blotting assays. **b** Western blotting was used to detect LC-3I, LC-3II, and p62 expression in HCT8 and HCT8Fu cells transfected with pcDNA3.1 or pcDNA-H19 and si-NC or si-H19, respectively. **c**, **d** Autophagosomes were observed by transmission electron microscopy in HCT8Fu and HCT8 cells transfected with si-NC or si-H19 and pcDNA3.1 or pcDNA-H19, respectively. For starvation, indicated cells were cultured in serum free Earle’s balanced salt solution (EBSS) medium as the positive control for observation of Autophagosomes. Bar scale, 2 μm. **e**, **f** LC3 aggregation in HCT8Fu cells (**e**) transfected with si-NC and si-H19 detected by the confocal microscope (Bar scale, 50 μm) and in HCT8 (**f**) transfected with pcDNA3.1 or pcDNA-H19 observed under the fluorescence microscope. Bar scale, 15 μm. **g** The cell sensitivity of HCT8 and HCT116 transfected with pcDNA3.1 or pcDNA-H19 to 5-Fu was evaluated using the MTT assay upon exposure to the step-up concentration of 5-Fu with or without CQ administration for 72h. **P<0.01, ***P<0.001. **h**, **j** Apoptosis was detected by flow cytometry in HCT8, HCT116 cells transfected with pcDNA3.1 or pcDNA-H19. The cells were treated with PBS or CQ. **i**, **k** Columns are the average of three independent experiments. Data are presented as mean±SD from three independent experiments. **P<0.01, ***P<0.001

The above results showed that H19 itself did not regulate apoptosis, which reminded us of the protective mechanism of cells under environmental pressure: autophagy. Several studies^23,24^ have reported that the protective autophagy can reduce apoptotic-cell death of CRC cells in response to 5-Fu treatment, and the inhibition of autophagy augments 5-Fu chemotherapy. We next hypothesized that H19 induced 5-Fu chemoresistance via autophagy pathway. To test the above hypothesis, we first assessed the LC3 protein cleavage level of CRC cell lines at the basal condition. HCT8 and HCT116 cells exhibited low basal condition. Comparatively, HCT8Fu and SW1116 cells exhibited high level (Fig. 4a). Western blotting showed that H19 significantly upregulated the formation of autophagy marker LC3-II. Moreover, another autophagy marker, SQSTM1, also known as p62 was repressed by H19 overexpression (Fig. 4b). Transmission electron microscopy showed an increase in the formation of autophagic vesicles in the H19 overexpressed HCT8 cells, and lesser autophagic vesicles in the H19 knocked down HCT8Fu cells (Fig. 4c, d). Autophagy inducer treatment (for starvation) with Earle’s balanced salt solution (EBSS) was used as the positive control. Immunofluorescence assay also showed that LC3 aggregation was significantly attenuated by si-H19 in HCT8Fu cells and promoted by overexpression of H19 in HCT8 cells (Fig. 4e, f). These results demonstrate that H19 can promote autophagy in CRC cells.

Furthermore, we treated HCT116 cells and HCT8 cells with pcDNA-H19 in the presence chloroquine (CQ), an autophagy lysosomal inhibitor. Addition of CQ could block the autophagic flux in the cells^23^. We found that H19-induced 5-Fu resistance was abolished by CQ, which was confirmed by MTT assay (Fig. 4g) and flow cytometric apoptosis analysis (Fig. 4h, j; untreated control was shown in Fig. SK and SL). Collectively, our date indicate H19 induces 5-Fu resistance via the autophagy pathway in CRC cells.

### H19 triggers autophagy via SIRT1

**Fig. 5:**
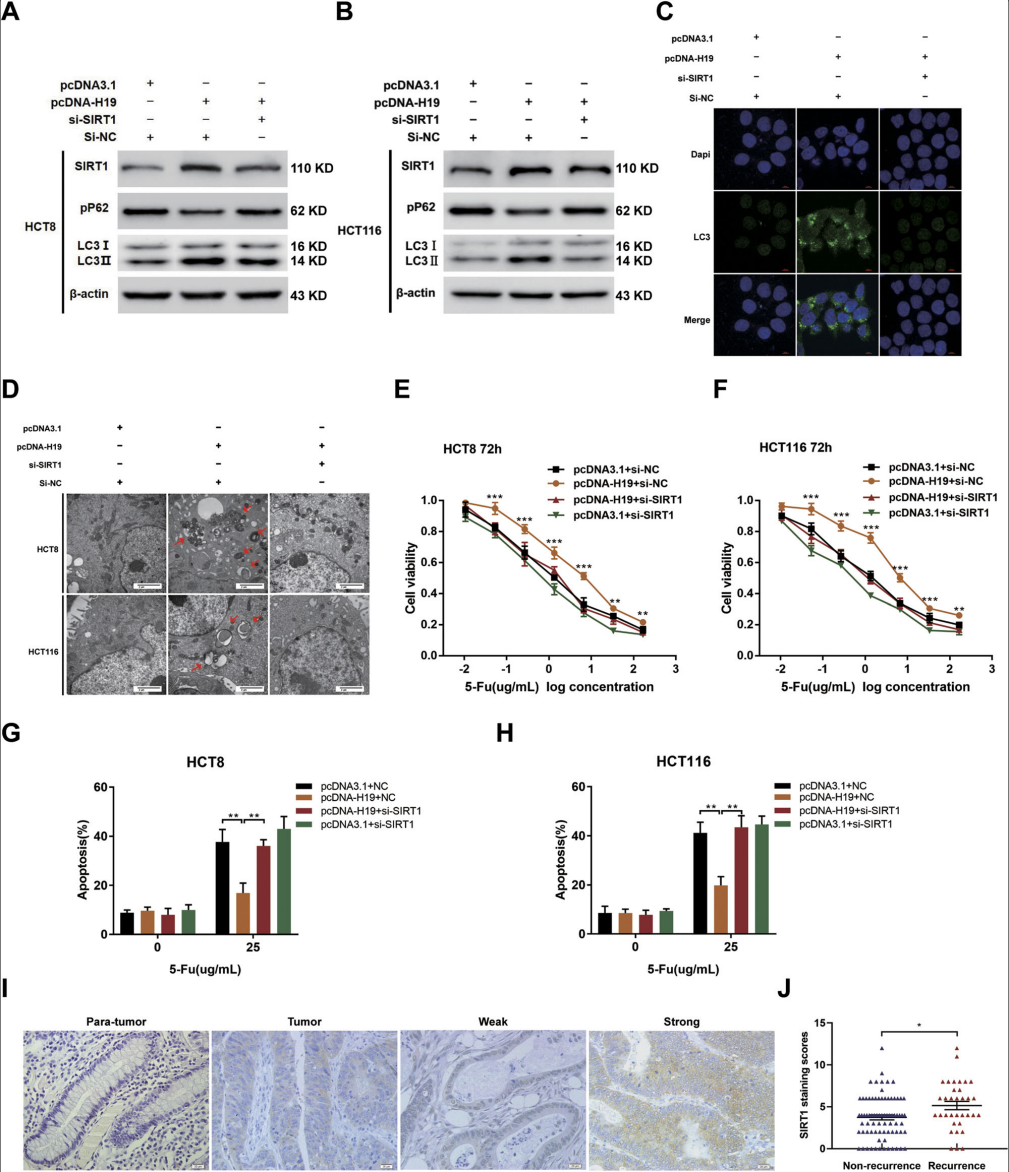
The H19/SIRT1/autophagy pathway attenuates the chemosensitivity of CRC cells. **a**, **b** Western blotting was used to detect LC-3I, LC-3II, p62, and SIRT1 expression in HCT8 and HCT116 cells transfected with pcDNA-H19 and/or si-SIRT1. **c** LC3 aggregation in HCT116 cells transfected with pcDNA-H19 and/or si-SIRT1. Bar scale, 10 μm. **d** Autophagosomes were observed by transmission electron microscopy in HCT8 and HCT116 cells transfected with pcDNA-H19 and/or si-SIRT1. Bar scale, 2 μm. **e**, **f** The cell sensitivity of HCT8 and HCT116 transfected with pcDNA3.1 or pcDNA-H19 to 5-Fu was evaluated using the MTT assay upon exposure to the step-up concentration of 5-Fu with or without downregulation of SIRT1 for 72h. **P<0.01, ***P<0.001. **g**, **h** Apoptosis was detected by flow cytometry in HCT8 (**g**) and HCT116 (**h**) cells transfected with pcDNA3.1 or pcDNA-H19 with or without downregulation of SIRT1 and treated with 5-Fu. **P<0.01. **i** Expression of SIRT1 was analyzed by IHC in the CRC tissues and paired adjacent normal samples. **j** IHC analysis was performed to determine the SIRT1 staining scores in CRC tissues with distinct recurrence status. *P<0.05

To examine whether autophagy elements or regulators such as ATG7 and SIRT1 may participate in H19-induced autophagy in CRC cells, western blotting was performed to analyze this protein expression status in pcDNA-H19 transfected CRC cells. Results showed that only the expression of SIRT1 was upregulated after pcDNA-H19 transfected both in HCT8 and HCT116 cells (Fig. SM). While previous reports have shown that SIRT1 contributes to autophagy through regulating different autophagy related proteins and pathways^25,26^. Therefore, we tested whether H19 mediated upregulation of SIRT1 protein can induce autophagy in CRC cells. As shown in Fig. 5a, b, overexpression of H19 enhanced LC3-II conversion and decreased p62 expression, which was remarkably alleviated by the silence of SIRT1 with the si-SIRT1–1 transfection (Fig. SN and SO; si-SIRT1–1 was used to silence SIRT1 in the subsequent experiments). Meanwhile, overexpression of H19 dramatically induced LC3 aggregation (Fig. 5c) and increased the autophagosomes (Fig. 5d) in CRC cells, which was markedly attenuated by the silence of SIRT1. All the above data indicated that H19 induces autophagy of CRC cells via up-regulating the expression of SIRT1. To uncover whether H19 exerts chemoresistance promoting functions in CRC by modulating SIRT1, we checked the effects of SIRT1 on H19-induced 5-Fu resistance and observed that SIRT1 knockdown blocked the H19-induced CRC cell 5-Fu resistance (Fig. 5e–h).

We next investigated whether SIRT1 participated in clinical implications of CRC. The expression of SIRT1 was detected by immunohistochemical analysis. Results showed that SIRT1 protein was located in both cytosol and nuclei of CRC cells (Fig. 5i). Besides, 34 recurrent CRC tissues from patients with recurrence had stronger SIRT1 expression compared with that of non-recurrence CRC tumor tissues (Fig. 5j). This phenomenon indicated that SIRT1 had underlying association with 5-Fu chemoresistance in CRC.

### H19 sponges miR-194–5p as ceRNA

**Fig. 6:**
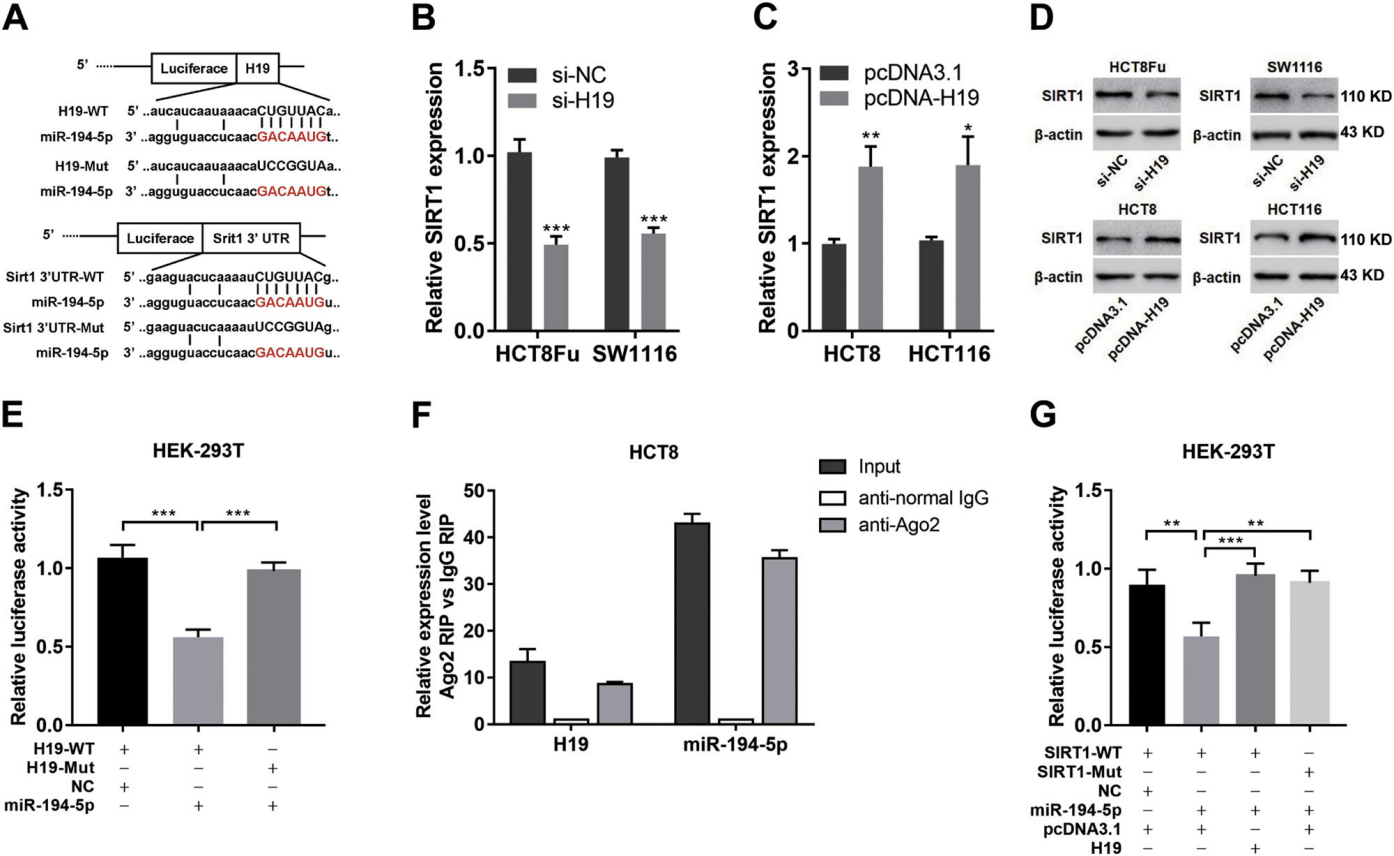
H19 sponges miR-194-5p as ceRNA. **a** Schematic illustration of the predicted-binding sites between H19 and miR-194-5p, SIRT1 and miR-194-5p, mutation of potential miR-194-5p binding sequence in H19 and SIRT1. **b**, **c** The mRNA expression levels of SIRT1 in HCT8Fu and SW1116 cells transfected with si-H19 (**b**) and HCT8 and HCT116 cells transfected with pcDNA-H19 (**c**) were analyzed by qRT-PCR. *P<0.05, **P<0.01, ***P<0.001. **d** The protein expression levels of SIRT1 in HCT8Fu and SW1116 cells transfected with si-H19 and HCT8 and HCT116 cells transfected with pcDNA-H19 were detected by western blotting. **e** The relative luciferase activity in HEK-293T cells with and without miR-194-5p overexpression was measured when transfected with pmiR-H19-WT or Mut luciferase plasmids using the dual-luciferase assay. ***P<0.001. **f** Cellular lysates from HCT8 cells were used for RIP with an anti-Ago2 antibody or IgG antibody. The levels of H19 and miR-194-5p were detected by qRT-PCR. **g** The relative luciferase activity in HEK-293T cells were cotransfected miR-194-5p and luciferase plasmid containing WT SIRT1 3'UTRs or Mut 3'UTRs with pcDNA-H19 or empty vector was determined using the dual-luciferase assay. **P<0.01, ***P<0.001

It has been reported that lncRNA can serve as a competing endogenous sponge RNA to interact with miRNAs and decrease the level of miRNA^9,27^. To explore the potential biological mechanism of H19 activity in SIRT1 expression, we used the starBase v2.0 and TargetScan to predict the potential-binding sites between H19 and miRNAs associated with SIRT1^28^. We found that H19 harbors a recognition sequence of miR-194–5p, and SIRT1 was a potential target gene of miR-194–5p (Fig. 6a). To determine whether SIRT1 was a potential target gene of H19, we transfected cells with pcDNA-H19 or si-H19 and then detected the expression of SIRT1. Results showed that H19 overexpression led to SIRT1 mRNA and protein levels increase at 72 h post transfection, and vice versa (Fig. 6b, d). To further confirm these, we manipulated H19 overexpression and then knocked it down. Results showed the recovery of SIRT1 level (Fig. SP and SQ).

Next, luciferase reporter containing the full length of H19 was constructed and the luciferase reporter assay was performed. We observed that miR-194–5p overexpression led to a marked inhibition in the reporter activity of pmiR-GLO-H19-WT compared with that of pmiR-GLO-H19-Mut (Fig. 6e), suggesting sequence specific binding and inhibition of H19 by miR-194–5p. To further validate the potential binding of H19 to miR-194–5p, RNA Immunoprecipitation (RIP) assay using an anti-Ago2 antibody was performed. The data exhibited that both H19 and miR-194–5p were obviously enriched in Ago2 complex, demonstrating that H19 is included in microRNA ribonucleoprotein complex (miRNP), probably through binding with miR-194–5p (Fig. 6f).

To determine whether SIRT1 is directly targeted by miR-194–5p at its 3′UTR, the luciferase reporter plasmid containing 3′UTR fragments of SIRT1 was cotransfected with miR-194–5p mimics and NC mimics. Luciferase activity assays showed that ectopic expression of miR-194–5p significantly decreased the luciferase activity of the WT- but not that of the Mut-SIRT1 3′UTR in HEK-293T cells (Fig. 6g). Besides, the overexpression of H19, not the vector control, blocked the inhibitory effect of miR-194–5p on the relative luciferase expression of pmiR-GLO-SIRT1–3′UTR. These results confirmed that H19 abolishes the miR-194-5p-mediated repressive activity on SIRT1 by competitively binding miR-194-5p.

### H19 sponges miR-194-5p to modulate SIRT1 expression

**Fig. 7:**
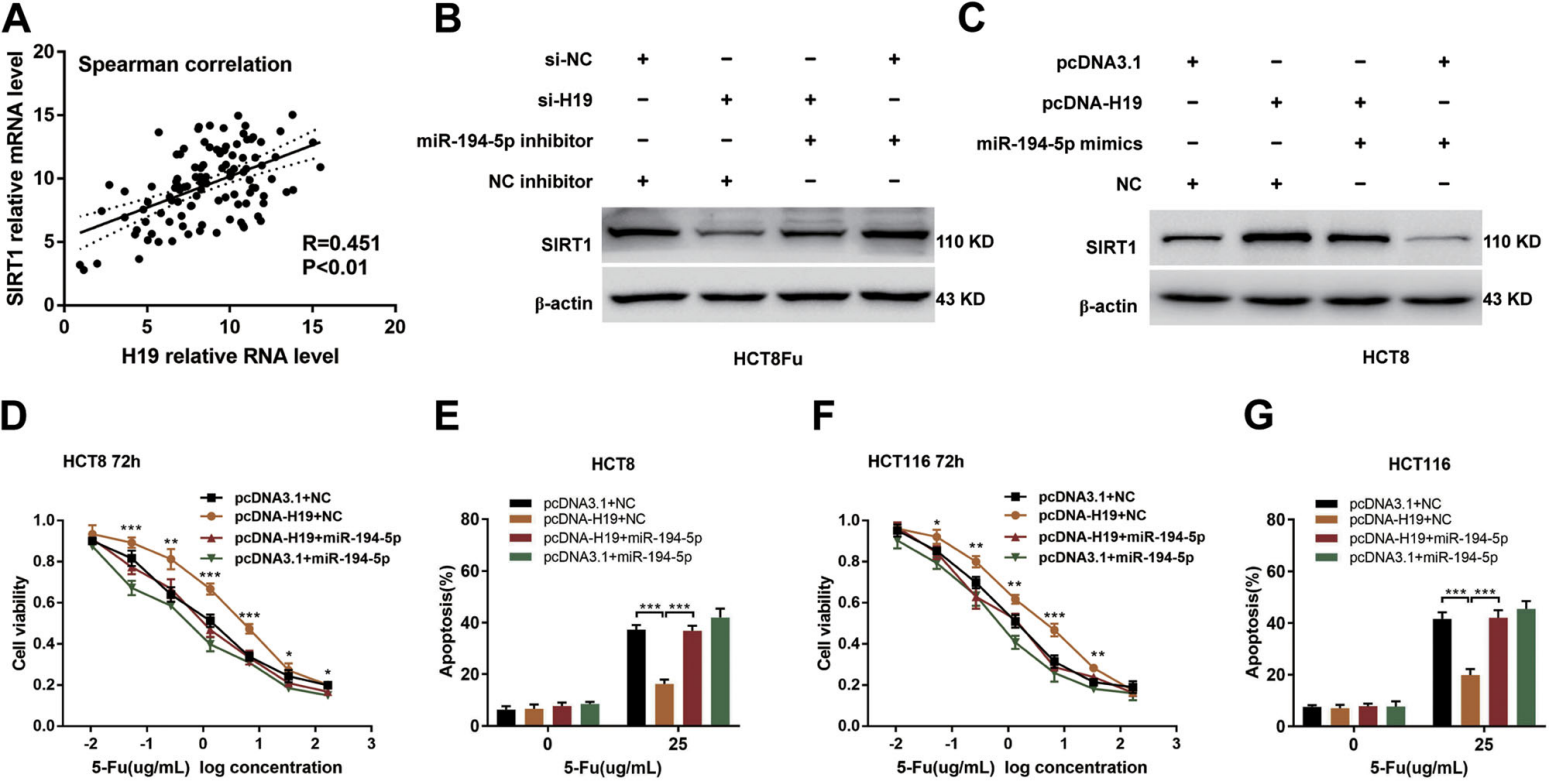
H19 sponges miR-194-5p to modulate SIRT1 expression. **a** CRC tumors were subject to qRT-PCR for detecting the correlation between H19 and SIRT1. **b**, **c** Western blotting was used to determine the SIRT1 expression level for si-H19 plus miR-194-5p inhibitor cotransfected into HCT8Fu cells, and pcDNA-H19 plus miR-194-5p mimics cotransfected into HCT8 cells. **d**, **f** The cell sensitivity of HCT8 (**d**) and HCT116 (**f**) transfected with pcDNA3.1 or pcDNA-H19 to 5-Fu was evaluated using the MTT assay upon exposure to the step-up concentration of 5-Fu with or without overexpression of miR-194-5p for 72h. *P<0.05, **P<0.01, ***P<0.001. **e**, **g** Apoptosis was detected by flow cytometry in HCT8 (**e**) and HCT116 (**g**) cells transfected with pcDNA3.1 or pcDNA-H19 with or without overexpression of miR-194-5p and treated with 5-Fu. ***P<0.001

**Fig. 8:**
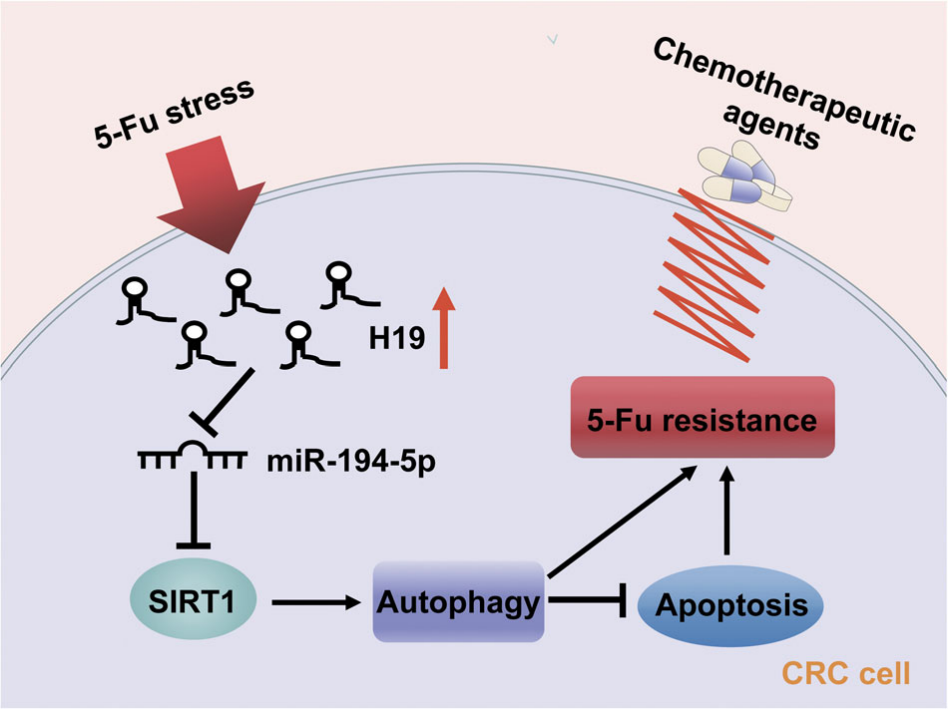
**The functional model underlying the mechanism of H19 on tumor chemoresistance**

According to the above discoveries, we assessed whether H19 correlated with SIRT1 expression in patients. For this purpose, qRT-PCR was performed to measure the expression of SIRT1 in the same cohort that was used to examine the expression of H19. Results showed that there was a positive correlation between H19 and SIRT1 expression in CRC tissues (Fig. 7a, *R* = 0.451, *P* = 0.0054). Intensively, results of the reversal experiment showed that while H19 down-regulation led to decreased expression of SIRT1, simultaneous miR-194-5p down-regulation was able to reverse the inhibition of SIRT1 expression in HCT8Fu resistant cell line (Fig. 7b), and vice versa in HCT8 sensitive cell line (Fig. 7c). These results confirmed that H19 abolishes the miR-194-5p-mediated repressive activity on SIRT1 by competitively binding miR-194-5p. We then evaluated the effects of miR-194-5p on the H19-induced 5-Fu resistance in CRC cells. As shown in Fig. 7d to  7g, ectopic miR-194-5p expression significantly reversed the H19-induced 5-Fu resistance and counteracted the apoptosis-inhibiting effects of H19 in CRC cells. Altogether, these data demonstrate that H19 exerts 5-Fu chemoresistance promoting functions in CRC, at least partly, through sponging miR-194-5p and then regulating SIRT1 dependent autophagy pathway (Fig. 8).

## Discussion

CRC is the leading cause of cancer deaths around the world and 5-Fu based chemotherapy has been widely used to treat different types of cancer including CRC. Understanding the mechanisms of chemoresistance in CRC is in an urgent need to improve the survival. lncRNAs are emerging from the “desert region” of the genome as a new source of biomarkers that could characterize disease recurrence and progression. H19 is reported to be associated with some types of carcinoma, and it is overexpressed and acts as an oncogene in breast cancer^29^, bladder cancer^18^, and chronic myeloid leukemia^30^. However, it still functions as a tumor-suppressor in hepatocellular cancer^31^. Our previous work revealed that H19 promotes tumor growth by recruiting and binding to eIF4A3^22^, indicating poor prognosis of colorectal cancer. In this study, we identified the significant up-regulation of H19 in CRC tissues from patients with recurrence. Importantly, Kaplan–Meier survival analysis showed the negative correlation of H19 expression level with RFS. Its predictive value was comparable to that of the AJCC stage. Thus, H19 may be a potential chemotherapeutic drug sensitivity marker for CRC patients.

To elucidate the underlying mechanism and find the novel therapeutic targets, drug-resistant cell lines were needed. In this study, we used HCT8Fu cells as acquired resistance to 5-Fu, which was developed by increasing doses of 5-Fu, and another cell line SW1116 was identified as being primary 5-Fu resistant. H19 was found up-regulated in both cell lines, and H19 knockdown sensitized the 5-Fu resistance while H19 overexpression improved the 5-Fu resistance, suggesting H19 might mediate drug resistance in CRC. In the further functional studies, we confirmed that H19 down or up-regulation altered IC50 value for 5-Fu in colorectal cancer cells,. Several studies have reported that H19 contributes to cisplatin^32^ and temozolomide^33^ resistance in other malignances. In the current study, however, we didn’t get similar results of H19 in the chemoresistance of CRC against oxaliplatin, which is also platinum-based drug used extensively for treating colorectal cancer, suggesting that versatile function of H19 in different type of cancer should be considered.

There is a lot of evidence that autophagic and apoptotic pathway crosstalk during cellular damage were induced by a variety of stressors^34^. Indeed, both processes can occur in the same cell, though often with different kinetics in which autophagy is utilized prior to the induction of apoptosis. In this model, we found that H19-induced 5-Fu resistance was abolished when autophagic flux was blocked by CQ, indicating H19 induces 5-Fu resistance via promoting autophagy and inhibiting apoptosis under 5-Fu stress. To elucidate the targets of autophagy, we screened a panel of autophagy elements and the results showed that H19 up-regulated SIRT1 expression at both the mRNA and protein level. SIRTs are a family of NAD^+^-dependent histone deacetylases. SIRT1 is the most extensively studied member among SIRT family^35^. The expression of SIRT1 is up-regulated in multiple types of tumors and is associated with tumor resistance to cancer chemotherapy. For example, studies showed that inhibiting SIRT1 sensitized pancreatic cancer cells to gemcitabine through induction of DNA damage and apoptosis^36^. Interestingly, some studies claimed that SIRT1 could inhibit tumor progression^37,38^. According to these, it remains controversial whether SIRT1 acts as an oncogene or tumor-suppressor, and its specific functions may depend on diverse biological signaling. In our present study, clinical samples showed that the mRNA expression level of SIRT1 was a positive correlation with that of H19. Simultaneously, we found that tissues from patients with recurrence had stronger SIRT1 expression compared with that of non-recurrence. These data indicated that SIRT1 performed as an oncogene in 5-Fu resistance mechanism of colorectal cancer.

Recent studies revealed a new mechanism of lncRNA by acting as ceRNA. It can block the repression of miRNA on its target by competitively binding their common miRNA responsive elements (MREs)^39^. In the current study, bioinformatics prediction was performed to clarify the underlying regulatory mechanism between H19 and SIRT1. Our results predicted miR-194-5p as a novel regulator of SIRT1. MiR-194-5p has been proved as a tumor regulatory factor in various cancers^40,41^. As expected, both luciferase and RIP analysis confirmed the binding of H19 to miR-194-5p. Results of the reversal experiment showed that while H19 down-regulation led to decreased expression of SIRT1, simultaneous miR-194-5p down-regulation was able to reverse the inhibition of SIRT1 expression in HCT8Fu resistant cell line. Furthermore, ectopic miR-194-5p expression significantly reversed the H19-induced 5-Fu resistance and counteracted the apoptosis-inhibiting effects of H19 in CRC cells. Altogether, these data demonstrate that H19 exerts 5-Fu chemoresistance promoting functions in CRC, at least partly in part, through sponging miR-194-5p and then regulating SIRT1 dependent autophagy pathway (Fig. 8).

In summary, our work shows that H19 is upregulated in CRC recurrence samples, correlated with patients’ survival and appears to be a potential biomarker for predicting 5-Fu chemoresistance. Our findings unveil that SIRT1 dependent autophagy pathway could affect 5-Fu chemoresistance in colon cancer cells, which was modulated by H19/miR-194-5p axis. Therefore, our study appears to support a tumor promoter role for SIRT1 in colorectal cancer. In addition to its biological importance, our work may be relevant in clinical management of CRC patients. As the amount of H19 is associated with the risk of CRC recurrence, the measurement of H19 post-surgery may be an effective approach to predict patients’ outcome. Furthermore, our data raise an important clinical question: are conventional chemotherapeutic regimens including 5-Fu suitable for CRC patients with a high amount of H19? Alternatively, we suggest that CRC patients with a high amount of H19 may be treated with conventional chemotherapy in combination with anti-H19 treatment and/or an autophagy inhibitor.

## Materials and methods

### Clinical samples

A total of 110 human CRC tissues and para-tumor tissues were collected in the Department of Colorectal Cancer Surgery, the Second Affiliated Hospital of Harbin Medical University. Patients were pathologically and clinically diagnosed with colorectal cancer. After surgical debulking, patients have undergone XELOX or mFOLFOX6 regimen therapy. Informed consent was obtained from the patients before sample collection in accordance with institutional guidelines. Recurrence was monitored by imaging examination systems (Chest X-ray and CT), gastrointestinal endoscopy with biopsy, and telephone follow-up. This study was carried out under the permission of the Clinical Research Ethics Committees of the Second Affiliated Hospital of Harbin Medical University. The characteristics of all patients see Supplementary Table S1.

### Cell lines

Human CRC HCT8 and HCT8Fu were purchased from Shanghai Meixuan Corporation (Shanghai, China) and were cultured according to a previous report^42^. SW1116 were purchased from Procell Life Science & Technology Co,.Ltd (Wuhan china). Human CRC cell lines (HCT116, HT29, Lovo, SW480, SW620) and a normal colon epithelium cell line (CCD-18Co) were purchased from the American Type Culture Collection (ATCC, Manassas, VA, USA), HCT116 and SW1116 were grown in Dulbecco’s modified Eagle’s medium (DMEM) or L15 medium (Gibco Laboratories, Grand Island, NY), and HEK-293T cells were grown in DMEM. All cells were cultured at 37 °C in a humidified incubator containing 5% CO2, supplemented with 10% fetal bovine serum (GIBCO, Carlsbad, CA).

### RNA extraction and qRT-PCR analyses

The total RNA was extracted from the tissues or cultured cells using TRIzol reagent (Invitrogen, Carlsbad, CA). Reverse transcribed complementary DNA was synthesized with random primers or microRNAs specific stem-loop primers. Subsequently, the cDNA was subjected to real-time PCR on a 7500 real-time PCR system (AB Applied Biosystems, Mannheim, Germany). Actin and U6 were used as internal controls. Primers sequences were listed in Supplementary Table S3.

### Cell transfection

Transfections were carried out using lipofectamine 2000 reagent (Invitrogen) according to manufactures instructions. Si-RNAs GenePharma (China) were used to knockdown gene expression. The vector pcDNA3.1-H19 was purchased from Santa Cruz (USA). MiR-194-5p mimics, NC (negative control) mimics, NC inhibitor and miR-194-5p inhibitor were purchased from GenePharma (China) (Supplementary Table S3).

### RNA immunoprecipitation (RIP)

RNA immunoprecipitation (RIP) experiments were performed using a Magna RIP™ RNA-Binding Protein Immunoprecipitation Kit (Millipore, USA) according to the manufacturer’s instructions. The antibodies used for RIP assays of AGO2 were obtained from Abcam Company.

### MTT assays

Exponentially growing cells were seeded at 10,000 cells (100μl culture medium) per well in 96-well plates and incubated for 12h. The cells were then exposed to different concentrations of 5-Fu, then 20μl of MTT (Sigma Chemicals, St. Louis, MO, USA; 5mg/ml in PBS) was added to each well, and the cells were cultured for an additional 4h. Subsequently, 200μl of DMSO was added to each well to dissolve the crystals. The values of the optical density at 490nm were then measured using a micro-plate reader.

### Flow cytometry

Apoptosis was examined by flow cytometric analysis. An Annexin V-FITC/PI double stain assay (BD Biosciences, San Jose, CA) was performed following the manufacturer’s protocol. The analysis was performed with FlowJo software (Treestar, Inc., San Carlos, CA). All the assays were performed in triplicate apoptosis analyses.

### Western blotting assay and antibodies

Western blotting Proteins were separated on a 10% sodium dodecyl sulfate-polyacrylamide gel electrophoresis (SDS-PAGE) and transferred onto a nitrocellulose membrane. The blots were incubated with antibody overnight at 4 °C. Following three washes, membranes were then incubated with secondary antibody overnight at 4 °C. Signals were visualized by ECL.

### Luciferase reporter assay

The DNA oligonucleotide and the pmiR-GLO Reporter Vector were used to build the luciferase report vectors (pmiR-H19-WT/pmiR-H19-Mut and pmiR-SIRT1-WT/pmiR-SIRT1-Mut). HEK-293 cells were used to measure luciferase activity. A Renilla luciferase-expressing plasmid pRL-TK (Promega) used as control was also cotransfected. Cells were harvested and luciferase activity was determined using the Dual Luciferase Reporter Assay Kit (Promega) at 24 h after transfection. The results are expressed as relative luciferase activity (firefly luciferase/Renilla luciferase).

### Immunofluorescence (IF) analysis

The cells were rinsed with PBS and fixed with 4% paraformaldehyde for 30 minutes at room temperature, followed by permeabilization with 0.1% sodium citrate plus 0.1% Triton X-100. The cells were subjected to immunofluorescent staining with antibody for 16 hours at 4 °C. The cells were then washed with cold PBS three times for five minutes each and incubated with fluorescence-labeled secondary antibody (1:400, #ZF0511, ZSGB-BIO) for 30min. The cells were visualized using microscope (FSX100, Olympus) or (C2+, NIKON).

### Statistical analysis

All statistical analyses were performed using SPSS 23.0 software (IBM). Data are expressed as the mean±SD for at least three separate experiments. Differences between groups were analyzed using the Student's *t*-test or one-way ANOVA. Overall survival was calculated by Kaplan–Meier survival analysis and compared using the log-rank test. *P*-values<0.05 were considered statistically significant.

## Electronic supplementary material

Supplementary figure S

Supplementary tables

Supplementary figure legends
