# Supplementary figures and images for: Long non-coding RNA H19 confers 5-Fu resistance in colorectal cancer by promoting SIRT1-mediated autophagy
Source: Cell Death Dis. 2018 Nov 19;9(12):1149. doi: 10.1038/s41419-018-1187-4 (PMC6242979; doi:10.1038/s41419-018-1187-4)

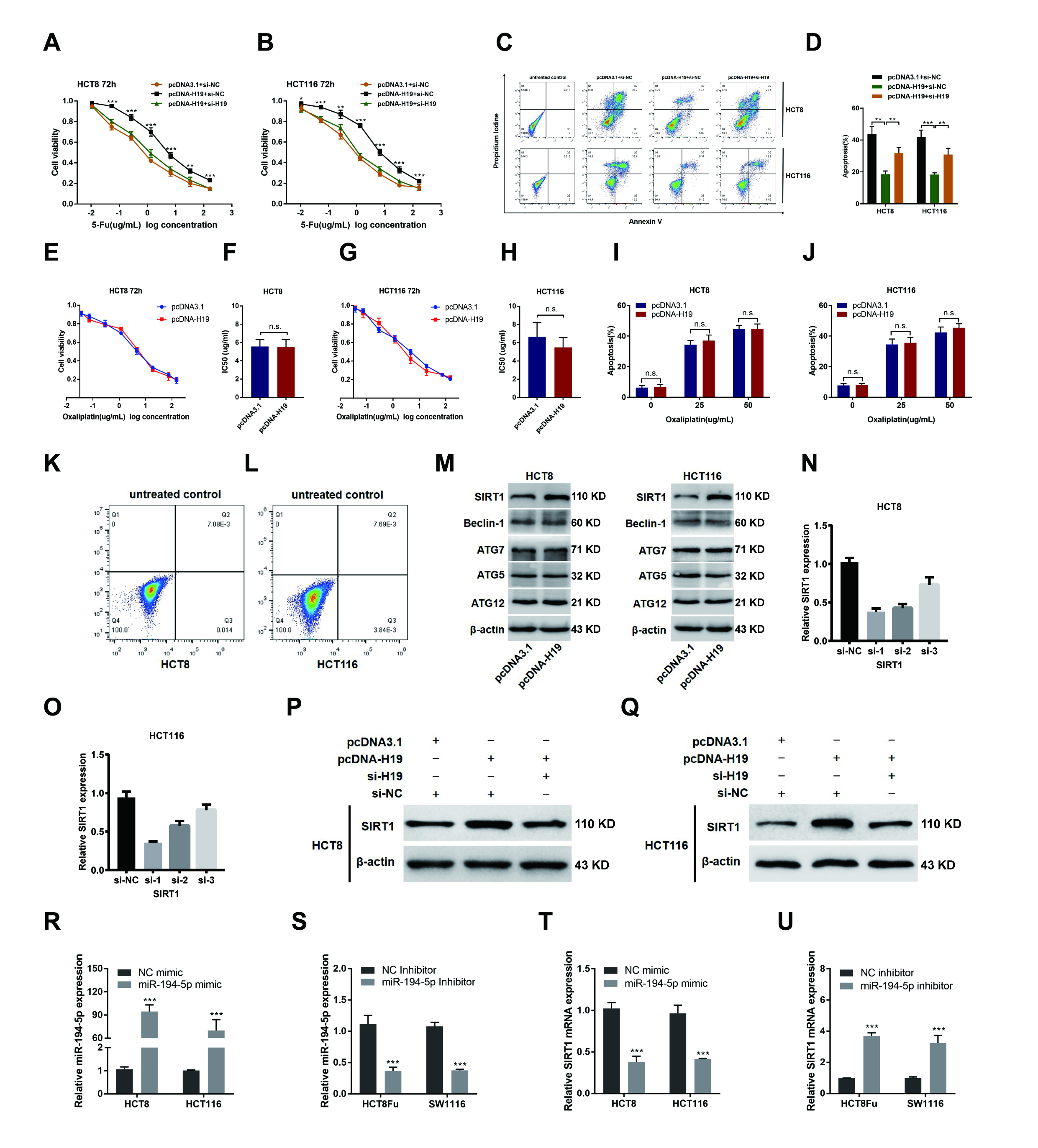

Supplement: Supplementary file 1 — Supplementary figure S [file 41419_2018_1187_MOESM1_ESM.tif]
