# Supplementary material for: Long non-coding RNA H19 confers 5-Fu resistance in colorectal cancer by promoting SIRT1-mediated autophagy
Source: Cell Death Dis. 2018 Nov 19;9(12):1149. doi: 10.1038/s41419-018-1187-4 (PMC6242979; doi:10.1038/s41419-018-1187-4)
Supplement: Supplementary file 2 — Supplementary tables [file 41419_2018_1187_MOESM2_ESM.docx]

| **Table S1. Characteristic of 110 patients with colorectal cancer** | | | |
| --- | --- | --- | --- |
| **Characteristics** | **No recurrence** | **Recurrence** | **p-value** |
|  | **n=76** | **n=34** |  |
| Age (years) |  |  |  |
| ＜60 | 43 | 24 | 0.164 |
| ≥60 | 33 | 10 |  |
| Gender |  |  |  |
| Male | 44 | 17 | 0.441 |
| Female | 32 | 17 |  |
| Tumor size |  |  |  |
| ＜5cm | 25 | 15 | 0.258 |
| ≥5cm | 51 | 19 |  |
| Location |  |  |  |
| Colon | 31 | 16 | 0.539 |
| Rectem | 45 | 18 |  |
| Differentiation |  |  |  |
| Well/Moderate | 64 | 27 | 0.538 |
| Poor | 12 | 7 |  |
| Depth of tumor |  |  |  |
| T1+T2 | 32 | 18 | 0.292 |
| T3+T4 | 44 | 16 |  |
| AJCC stage |  |  |  |
| Ⅱ | 41 | 9 | 0.007 |
| Ⅲ | 35 | 25 |  |
| H19 |  |  |  |
| Low | 44 | 11 | 0.013 |
| High | 32 | 23 |  |

**Table S2. Correlation of expression of H19 with clinicopathologic features**

| **Characteristics** | **H19** | | ***p-***value |
| --- | --- | --- | --- |
|  | **Low** | **High** |  |
| Age (years) |  |  |  |
| ＜60 | 33 | 34 | 0.845 |
| ≥60 | 22 | 21 |  |
| Gender |  |  |  |
| Male | 32 | 29 | 0.565 |
| Female | 23 | 26 |  |
| Tumor size |  |  |  |
| ＜5cm | 17 | 23 | 0.234 |
| ≥5cm | 38 | 32 |  |
| Location |  |  |  |
| Colon | 22 | 25 | 0.563 |
| Rectem | 33 | 30 |  |
| Differentiation |  |  |  |
| Well/Moderate | 50 | 41 | 0.023 |
| Poor | 5 | 14 |  |
| Depth of tumor |  |  |  |
| T1+T2 | 32 | 18 | 0.007 |
| T3+T4 | 23 | 37 |  |
| AJCC stage |  |  |  |
| Ⅱ | 26 | 24 | 0.702 |
| Ⅲ | 29 | 31 |  |

**TABLE S3 Primer for qRT-PCR and oligonucleotides**

| **Primer or oligonucleotide specificity** | **Direction^a^** | **Sequence** |
| --- | --- | --- |
| **Primers** |  |  |
| miR-194-5p stem-loop |  | GTCGTATCCAGTGCGTGTCGTGGAGTCGGCAATTGCACTGGATACGACTCCACA |
| miR-194-5p | F | CGGGCTGTAACAGCAACTCCA |
|  | R | ATTGCGTGTCGTGGAGTCG |
| beta Actin | F | TGGCACCCAGCACAATGAA |
|  | R | CTAAGTCATAGTCCGCCTAGAAGCA |
| SIRT1 | F | TAGCCTTGTCAGATAAGGAAGGA |
|  | R | ACAGCTTCACAGTCAACTTTGT |
| U6 | F | GCTTCGGCAGCACATATACTAAAAT |
|  | R | CGCTTCACGAATTTGCGTGTCAT |
| H19 | F | TGCTGCACTTTACAACCACTG |
|  | R | ATGGTGTCTTTGATGTTGGGC |
| **Oligonucleotides** |  |  |
| miR-194-5p mimic | S | UGUAACAGCAACUCCAUGUGGA |
|  | A | CACAUGGAGUUGCUGUUACAUU |
| NC | S | UUCUCCGAACGUGUCACGUTT |
|  | A | ACGUGACACGUUCGGAGAATT |
| miR-194-5p inhibitor |  | UCCACAUGGAGUUGCUGUUACA |
| NC inhibitor |  | CAGUACUUUUGUGUAGUACAA |
| si-H19 | S | CCAGGCAGAAAGAGCAAGATT |
|  | A | UCUUGCUCUUUCUGCCUGGTT |
| si-SIRT1-1 | S | GCAACUAUACCCAGAACAUTT |
|  | A | AUGUUCUGGGUAUAGUUGCTT |
| si-SIRT1-2 | S | GCUGAUGAACCGCUUGCUATT |
|  | A | UAGCAAGCGGUUCAUCAGCTT |
| si-SIRT1-3 | S | CCAAGCAGCUAAGAGUAAUTT |
|  | A | AUUACUCUUAGCUGCUUGGTT |

^a^ F, forward; R, reverse; S, sense; A, antisense.

**Table S4 Reagents or resources**

| **Reagents or resources** | **Source** | **Identifier** |
| --- | --- | --- |
| Antibodies |  |  |
| Anti-Argonaute-2 | Abcam | Cat#ab32381 |
| Anti-SQSTM1/P62 | Abcam | Cat#ab56416 |
| Anti-SIRT1 | Abcam | Cat#ab32441 |
| Anti-Thymidylate Synthase | Abcam | Cat#ab58287 |
| Anti-Beclin1 | Cell Signaling Technology | Cat#4122s |
| Anti-Cleaved Caspase-3 | Cell Signaling Technology | Cat#9661 |
| Anti-Caspase-3 | Cell Signaling Technology | Cat#9662 |
| Anti-LC3A-B | Cell Signaling Technology | Cat#12741s |
| Anti-PARP | Cell Signaling Technology | Cat#9532 |
| Anti-ATG7 | Wanleibio | Cat#WL02793 |
| Anti-ATG5 | Wanleibio | Cat#WL02411 |
| Anti-ATG12 | Wanleibio | Cat#WL03144 |
| beta Actin | Genscript | Ca#A00702 |
| Chemicals |  |  |
| 5-Fluorouracil | Sigma | F6627 |
| Oxaliplatin | sigma | O9512 |
| Chloroquine | Sigma | C6628 |
| EBSS | Solarbio | H2045 |
