# Supplementary material for: Long non-coding RNA H19 confers 5-Fu resistance in colorectal cancer by promoting SIRT1-mediated autophagy
Source: Cell Death Dis. 2018 Nov 19;9(12):1149. doi: 10.1038/s41419-018-1187-4 (PMC6242979; doi:10.1038/s41419-018-1187-4)
Supplement: Supplementary file 3 — Supplementary figure legends [file 41419_2018_1187_MOESM3_ESM.docx]

**Supplementary Figure S**

(A, B) The cell sensitivity of HCT8 (A) and HCT116 (B) transfected with pcDNA3.1 or pcDNA-H19 to 5-Fu was evaluated using the MTT assay upon exposure to the step-up concentration of 5-Fu with or without subsequently knocking down H19 expression. **P* < 0.05, ***P* < 0.01, ****P* < 0.001. (C) Apoptosis was detected by flow cytometry in HCT8 and HCT116 cells transfected with pcDNA3.1 or pcDNA-H19 with or without subsequently knocking down H19 expression and treated with 5-Fu (25μg/ml). (D) Columns are the average of three independent experiments. Data are presented as mean ± SD from three independent experiments. ***P* < 0.01, ****P* < 0.001. (E, G) The cell sensitivity of HCT8 (E) and HCT116 (G) cells transfected with pcDNA3.1 or pcDNA-H19 to oxaliplatin was evaluated using the MTT assay upon exposure to the step-up concentration of oxaliplatin for 72 h. (F, H) The IC50 value of HCT8 (F) and HCT116 (H) cells in the pcDNA3.1 or pcDNA-H19 group was calculated. n.s. nonsignificant. (I, J) Apoptosis was detected by flow cytometry in HCT8 (I) and HCT116 (J) cells transfected with pcDNA3.1 or pcDNA-H19 and treated with different concentration of oxaliplatin. n.s. nonsignificant. (K, L) Flow cytometric apoptosis analysis of untreated control. (M) Western blotting was performed to detect autophagy-related protein expression in HCT8 cells (left) and HCT116 cells (right) transfected with pcDNA3.1 or pcDNA-H19. (N, O) Validation of knockdown efficacy of SIRT1 in HCT8 and HCT116 cells by qRT-PCR. (P, Q) HCT8 (P) and HCT116 (Q) transfected with pcDNA3.1 or pcDNA-H19 were detected by Western blotting with or without subsequently knocking down H19 expression. (R) HCT8 and HCT116 cells were transfected with miR-194-5p mimic (or NC mimic). The expression of miR-194-5p was validated by qRT-PCR. ****P* < 0.001. (S) HCT8Fu and SW1116 were transfected with inhibitor (or NC inhibitor). The expression of miR-194-5p was validated by qRT-PCR. ****P* < 0.001. (T, U) qRT-PCR analysis was applied to detect mRNA of SIRT1 in HCT8 and HCT116 cells. ****P* < 0.001. Data are presented as mean ± SD from three independent experiments.
